# The Neuroprotective Potential of Endophytic Fungi and Proposed Molecular Mechanism: A Current Update

**DOI:** 10.1155/2022/6214264

**Published:** 2022-10-01

**Authors:** Prabhakar Semwal, Sakshi Painuli, Jigisha Anand, Natália Cruz Martins, Marisa Machado, Rohit Sharma, Gaber El-Saber Batiha, Clement Amen Yaro, Jose M. Lorenzo, Md. Mominur Rahman

**Affiliations:** ^1^Department of Life Sciences, Graphic Era Deemed to be University, Dehradun (248002), Uttarakhand, India; ^2^Uttarakhand Council for Biotechnology (UCB), Prem Nagar, Dehradun (248007), Uttarakhand, India; ^3^Department of Biotechnology, Graphic Era Deemed to be University, Dehradun (248002), Uttarakhand, India; ^4^Institute of Research and Advanced Training in Health Sciences and Technologies (CESPU), Rua Central de Gandra 1317, Gandra PRD 4585-116, Portugal; ^5^TOXRUN-Toxicology Research Unit, University Institute of Health Sciences, CESPU, CRL, Gandra 4585-116, Portugal; ^6^Faculty of Medicine, University of Porto, Alameda Prof. Hernani Monteiro, Porto 4200-319, Portugal; ^7^Institute for Research and Innovation in Health (i3S), University of Porto, Porto 4200-135, Portugal; ^8^Department of Rasashastra and Bhaishajya Kalpana, Faculty of Ayurveda, Institute of Medical Sciences, Banaras Hindu University, Varanasi, India; ^9^Departments of Pharmacology and Therapeutics, Faculty of Veterinary Medicine, Damanhour University, Damanhour, Egypt; ^10^Department of Animal and Environmental Biology, University of Uyo, Uyo, Akwa Ibom, Nigeria; ^11^Centro Tecnológico de la Carne de Galicia, Parque Tecnológico de Galicia, Rúa Galicia No. 4, San Cibrao Das Viñas 32900, Spain; ^12^Department of Pharmacy, Faculty of Allied Health Sciences, Daffodil International University, Dhaka 1207, Bangladesh

## Abstract

Millions of people are affected by neuronal disorders that are emerging as a principal cause of death after cancer. Alzheimer's disease, ataxia, Parkinson's disease, multiple system atrophy, and autism comprise the most common ones, being accompanied by loss of cognitive power, impaired balance, and movement. In past decades, natural polyphenols obtained from different sources including bacteria, fungi, and plants have been utilized in the traditional system of medicine for the treatment of several ailments. Endophytes are one such natural producer of secondary metabolites, namely, polyphenols, which exhibit strong abilities to assist in the management of such affections, through modifying multiple therapeutic targets and weaken their complex physiology. Limited research has been conducted in detail on bioactive compounds present in the endophytic fungi and their neuroprotective effects. Therefore, this review aims to provide an update on scientific evidences related to the pharmacological and clinical potential along with proposed molecular mechanism of action of endophytes for neuronal protection.

## 1. Introduction

Neurodegenerative diseases (ND) include debilitating conditions that pose a serious threat to the human health leading to progressive degeneration of nerve cells. The brain disorders like Alzheimer's disease (AD), ataxia, Parkinson's disease (PD), multiple system atrophy, autism, are significantly linked to insufficient production of neurotransmitters, abnormal ubiquitination, aggregation of abnormal proteins followed by inflammation, and oxidative stress in the central nervous system (CNS) [1–3]. Although, there has been progression in our understanding about ND, the potential triggers of such disorders and their molecular mechanisms are still uncertain [1, 3]. Currently, no reliable cure is being available for the treatment of ND due to limited regeneration ability of CNS [4, 5]. The commercially available therapies are generally symptomatic and are recommended to alleviate the manifestation of disease and also to improve the health status of the patient's life. Furthermore, the treatment includes synthetic neuro medicines, associated with severe side effects [1, 6, 7]. A plethora of evidence has indicated promising therapeutic potential of natural bioactive compounds including various classes like phenols, flavonoids, alkaloids, and terpenoids, with high antioxidant activity against ND [8–13].

Endophytes are an endosymbiotic class of microorganisms, majorly comprising of bacteria and fungi colonizing in the tissues of healthy plants without posing any detrimental effect to their host. They are the treasure house of secondary metabolites such has flavonoids, alkaloids, polyphenols, saponins, and tannins with multiple therapeutic benefits [14–17]. Their richness in bioactive compounds make them fruitful candidates for drug development against different disorders, such as cancer, diabetes, hypertension, cardiovascular, gastrointestinal, and ND [18–23]. Among bacterial endophytes, there are more than 200 genera of bacterial species including *Streptomyces, Agrobacterium, Acinetobacter, Bacillus, Pseudomonas, Xanthomonas, Brevibacterium,* and *Microbacterium*, which are considered to synthesize metabolites with known antimicrobial and antioxidant activity [24–26]. Endophytic fungi are considered as a good source of antibiotics and anticancer drugs extracted from *Penicillium*, *Fusarium* spp., *Pestalotiopsis jesteri,* C*hloridium* spp., *Beauveria bassiana*, and *Metarhizium anisopliae* [27–29]. Series of bioactive chemical compounds have been isolated from endophytes, investigation has revealed their medicinal activity in several disease models and therefore could be an excellent source of drug for antibacterial, antiviral, antifungal, anticancer, anti-inflammatory, and neuroprotective purposes [30–33]. With ongoing scientific studies, there is a hope of a finding multipotential role of novel endophytic bioactive molecules against several health impairments, including neurodegenerative disorders [7, 34–36]. The present review will discuss the updated and quantified information on bioactive compounds of endophytic fungi and their effects on different ND with promising pharmacological or clinical perspectives.

## 2. Methodology

Published literature on the neuroprotective potential of endophytic fungi were collected from different online sources such as PubMed, ScienceDirect, Web of Science, SpringerLink, Wiley online library, and Google Scholar by using specific keywords “*Neuroprotective activities of endophytic fungi*” and “*Bioactive compounds of endophytic fungi and neuroprotection*” from 2006 to 2022 (July). Published research and review articles, and book chapters in English were included in this study, whereas duplicate and inappropriate articles related with the topic were excluded from the study.

## 3. Endophytic Fungi as a Source of Bioactive Compounds

**Figure 1:**
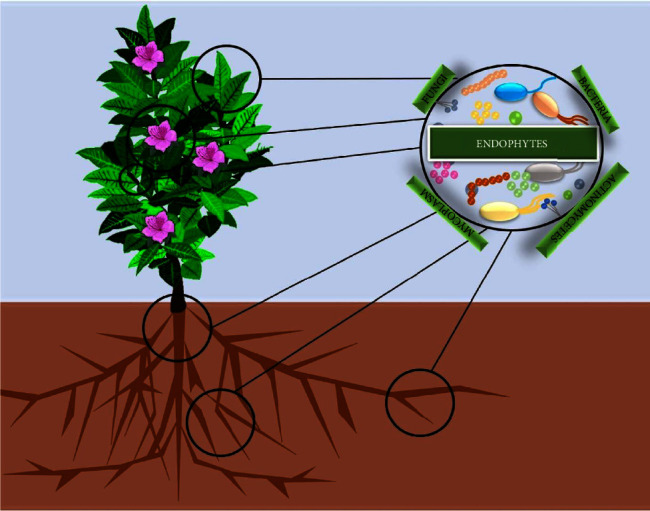
Endophytes residing in intercellular or intracellular locations in the plants.

For centuries, human civilization has greatly depended on plant sources in drug formulations to fight against numerous forms of diseases. Various plant species serve as a major resource for the isolation of diverse active compounds including, alkaloids, phenols, flavonoids, and vitamins, which act on diseases like cancer, diabetes, microbial diseases, neurological disorders, heart diseases, and skin disease [37–40]. However, in the international market, the demand for active compounds is continuously increasing due to which many plant species are facing severe threats. This problem raises an increased interest among worldwide researchers to find other alternative sources for extraction of the high valued secondary metabolites. In the last few decades, it has been reported that microorganisms integrated with plants, also known as endophytes, can synthesize biologically active compounds which possess promising therapeutic potential [14]. Generally, endophytes are class of microorganisms often actinomycetes, bacteria, and fungi which resides in intercellular or intracellular locations in the plants and show endosymbiotic association with the host plant (Figure 1) [41, 42].

**Figure 2:**
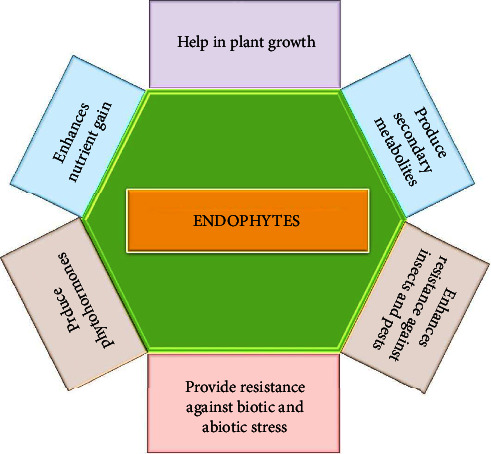
Different activities of endophytes in plant growth and development.

They play a significant role in synthesis of novel biologically active compounds including phenols, quinones, alkaloids, saponins, tannins, and flavonoids [43]. These microorganisms are found in almost all plant species, are ubiquitous in nature, and show complex interactions (antagonism, rarely parasitism, and mutualism) with host plants [44]. Endophytes help plants in many ways like enhancing the plant growth and nutrient uptake from the surrounding (Figure 2). They are known to colonize different plant parts including leaf segments, fruits, roots, stems, buds, seeds, petioles, inflorescence, and also in deceased and hollow plant cells [45–47].

**Figure 3:**
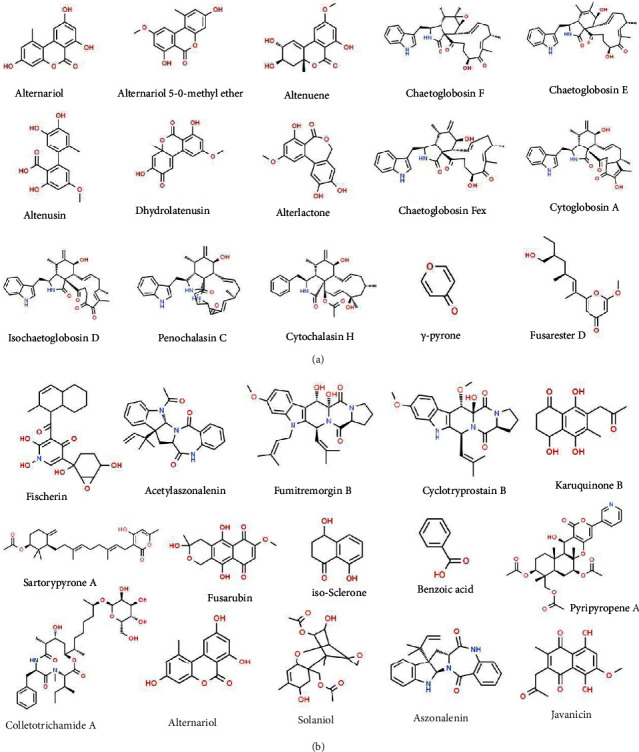
(a) Bioactive compounds previously isolated from endophytic fungi. Figure 3 (b) Bioactive compounds previously isolated from endophytic fungi.

Fungi are an important group of heterotrophic organisms which have complex lifecycle with multiple stages and interestingly they are observed to have a symbiotic relationship with autotrophs. They are also referred as symptomless symbionts which reside within the plant tissues of angiosperms, gymnosperms, ferns, and mosses [27, 48]. According to life history and phylogeny, endophytic fungi are grouped into two: clavicipitaceous and nonclavicipitaceous. Clavicipitaceous endophytic fungi are restricted to cool regions and cause infection in some grasses; however, nonclavicipitaceous are confined to the Ascomycota or Basidiomycota group and are present in vascular and nonvascular plant tissues [49, 50]. Endophytic fungi help host plants in nutrient uptake, produce plant growth hormones like auxins, gibberellins, and cytokinins, and aid plants in enhancing their self-defense mechanism [49, 51]. The active compounds generated by these fungi are essential for determining the adaptability of both the endophytic fungi and their host plant, especially in harsh environmental conditions, which include biotic and abiotic stresses [52–57]. Also, the bioactive compounds by these fungi possess potential applications in the food, cosmetic, agriculture, and medicine industries [58]. Pestalotheol C, an antiviral compound is isolated from *Pestalotiopsis theae*, an endophytic fungus [59]. Phomopsichalasin, an antibacterial compound which shows significant bactericidal activity against human pathogenic Gram-positive and Gram-negative bacteria, is obtained from *Phomopsis* sp. and plant host *Salix gracilistyla* [60, 61]. Anticancer and antineoplastic agents such as taxol, vincristine, vinblastine, and camptothecin can be isolated from the endophytic fungus *Taxomyces andreanae*, *Alternaria* spp., *Fusarium oxysporum,* and *Entrophospora infrequens* [62–65]. Subglutinol A and Aspernolide compounds used as immunosuppressive and cardio-protective agents are isolated from the endophytic fungus *Fusarium subglutinans* and *Aspergillus terreus* [66, 67]. Few neuroprotective agents including sanguinarine, isofraxidin, and vitexin have been isolated from endophytic sources [43]. However, much concern is needed in exploring more bioactive compounds from endophytic fungus. There is a need for more efforts in conducting clinical trials and applications that will help in developing the high-quality therapeutic agents. Some bioactive compounds present in endophytic compounds are alternariol, alternariol 5-O-methyl ether, alternuene, chaetoglobosin F, chaetoglobosin E, alternusin, dehydroalternusin, alterlactone, chaetoglobosin fex, cytoglobosin A, isochaetoglobosin D, penochalasin S, cytochalasin H, T-pyrone, fusarester D, fischerin, acetylaszonalenin, fumitremorgin B, cyclotryprostatin B, karuquinone B, sartorypyrone A, fusarubin, iso-sclerone, benzoic acid, pyripyropene A, colletotrichamide A, solaniol, aszonalenin, and javanicin (Figure 3(a) and 3(b)).

## 4. Neuroprotective Studies and Proposed Molecular Mechanism

Neurodegeneration is defined as a slow and progressive loss of neuronal structure and function in the specified region of the brain that resulted in neuronal cell death [68, 69]. By 2040, the ND are estimated to exceed cancer in ranking, as the second major cause of death among the elderly [70]. Therefore, it is important to explore therapeutic compounds from natural resources against ND as they possess higher benefits including no/fewer side effects, cost effective, and easily available, over synthesized compounds. Neuroprotective effects of different bioactive compounds isolated from endophytic fungi have been investigated for cure and management of neurodegenerative diseases. This review highlights the endophytes-derived bioactive compounds and their proposed mechanism of action via different pathways with therapeutic applications.

Recently, bioactive compounds present in endophytic fungi *Nigrospora oryzae* were screened for their acetylcholinesterase (AChE) and antioxidant activity [71]. Also, one of the isolates from the study, *Nigrospora oryzae* (GL15) showed maximum AChE as well as antioxidant activity, and the compound (fraction 3) accountable for these activities was identified as quercetin based on analyses using ultra-violet spectrophotometers (UV), fourier-transform infrared spectroscopy (FTIR), electrospray ionisation mass spectrometry (ESI-MS), high-performance liquid chromatography, (HPLC) and proton nuclear magnetic resonance (^1^H NMR). Additionally, the extract exhibited antidementia-like activity which led to learning and memory shortfalls through the AChE-mediated mechanism in the scopolamine model. The extract also enhanced the scopolamine-induced modulation in the cholinergic pathway and as well as triggered decrease in the activity of AChE and restoration of cyto-architecture of hippocampus [71]. While in another study by Hou and group [72], a total of seven dibenzopyrone phenolic derivatives including alternariol, alternariol 5-O-methyl ether, altenusin B, altenuene, altenusin, alterlactone, and dehydroaltenusin were extracted and identified using different spectroscopic methods from the endophytic fungi, *Alternaria alternate*. In this study, the compounds altenuene, altenusin, alterlactone, and dehydroaltenusin demonstrated significant neuroprotective effects against oxidative injuries by acting as potent activators of nuclear factor-erythroid derived 2-like 2 in PC12 cells. These compounds induced the nuclear accumulation of Nrf2, promoted the expression of Nrf2-governed cytoprotective genes, as well as increased the cellular antioxidant capacity [72]. Al-Qaralleh [73] in their study evaluated the crude extract *of Fusarium* spp., an endophytic fungi, and isolated OQ-Fus-2-F collected from the stem of *Euphorbia* plant. The crude extracts were tested for biological activities including antibacterial, antioxidant, and AchE inhibitory activity. The isolate OQ-Fus-2-F showed moderate biological activity in terms of antioxidant activity (ABTS : IC_50_ = 37.5 ± 3.5 *µ*g/mL and DPPH : IC_50_ = 191.3 ± 17.6 *µ*g/mL) and AChE inhibition activity (IC_50_ = 177.0 ± 13.7 *µ*g/mL), respectively [73].

A total of eight compounds, namely, chaetoglobosin F, chaetoglobosin fex, chaetoglobosin E, cytoglobosin A, penochalasin C, isochaetoglobosin D, cytochalasin H, and 18-methoxycytochalasin J were isolated from two endophytic fungi, *Chaetomium globosum* and *Phomopsis spp*. [74]. The antioxidant and neuroprotective activities of these isolated compounds were evaluated. Among all these compounds, chaetoglobosin, isochaetoglobosin, and cytochalasin showed significant antioxidant potential in DPPH (EC_50_ = 0.002 ± 0.001 mmol/L, 0.002 ± 0.001 mmol/L, 0.002 ± 0.001 mmol/L) and ABTS (0.002 ± 0.004 mmol/L, 0.002 ± 0.001 mmol/L, 0.001 ± 0.001 mmol/L) assays when compared with control (Vitamin E : EC_50_ = 0.079 ± 0.001 mmol/L, EC_50_ = 0.718 ± 0.008 mmol/L). These compounds also inhibited the H_2_O_2_/MMP^+^ and induces damage in PC12 cells by increasing cell viability and as well as decreasing the release of lactate dehydrogenase [74].

Lee et al. [75] isolated tricyclic pyridine alkaloids including (1) 6-deoxyoysporidinone (SSF2-1), (2) 4,6′-anhydrooxysproridinone (SSF2-2), and (3) sambutoxin (SSF2-3) from *Fusarium lateritium* (SSF2). Furthermore, SSF2-1, SSF2-2, and SSF2-3 were evaluated for their protective effects against glutamate-induced HT22 cell death. The compound SSF2-2 showed the significant protective effects against HT22 cells from cytotoxicity induced by glutamate, it reduces the intracellular accumulation of ROS, increases in superoxide anion production, Ca^2+^ influx, and depolarization of mitochondrial membrane potential. Additionally, the compound SSF2-2 increased the expression of Nrf2 and HO^−1^ pathways, whereas inhibited the apoptotic cell death via inhibition of cytochrome c and cleaved caspase-9, -3 in glutamate-induced HT22 cells [75]. Choi and co-workers, isolated and identified six neuroprotective bioactive compounds present in an endophytic fungi *Fusarium solani* JS-0169 collected from the leaves of *Morus alba* [76]. These six bioactive compounds, namely, ϒ-pyrone, fusarester D, karuquinone B, javanicin, solaniol, and fusarubin were identified via NMR spectroscopy analysis. These compounds showed protective activity against glutamate-induced cytotoxicity in HT22 cells. Among these compounds, ϒ-pyrone, javanicin, and fusarubin showed the acceptable neuroprotective activity in a dose-dependent manner. However, fusarubin at 12.5 *µ*M concentration displayed highest cell viability of 90.7 ± 4.5% in HT22 cells, it also possess strong DPPH scavenging activity [76].

A research group from China isolated and identified a total 26 endophytic fungi from the leaves, stems, and roots of the wild *Huperzia serrate.* Among these fungi, *Fusarium verticillioides, Fusarium oxysporum, Mucor racemosus, Mucor fragilis,* and *Trichoderma harzianum* produce Huperzine A, a potent AChE inhibitor against AD, using thin layer chromatography (TLC), HPLC, and LC-MS/MS analyses [77]. However, in another study, Zaki and co-workers from Egypt also isolated and identified some endophytic fungi from the different parts of wild *Huperzia serrata,* which were evaluated for their anti-AChE activity and Huperzine A production [78]. However, among all 11 isolates (AGF040 to AGF050), only four endophytic fungal isolates (AGF041, 42, 44, and 46) of *Alternaria spp., Penicillium spp.,* and *Colletotrichum spp.* genera displayed AChE inhibition activity (more than 50%) however, endophytic fugal isolate *Alternaria brassicae* AGF041, demonstrated the maximum inhibitory activity (75.5 ± 0.5%), and Huperzine A production, respectively [78].

**Figure 4:**
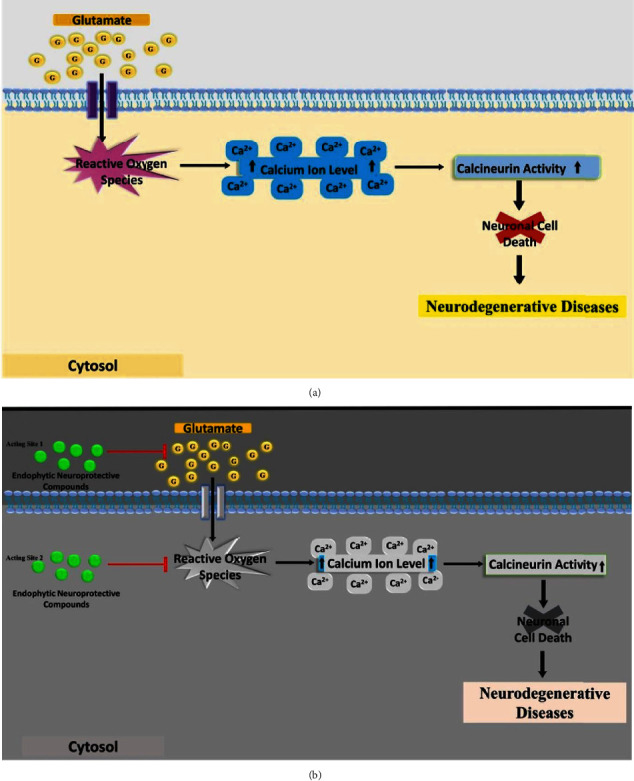
Proposed molecular mechanism of neuroprotection from compounds isolated from endophytic fungi. (a) Glutamate at higher level can cause neuronal cell death and cause neurodegenerative disorders; (b) Endophytic neuroprotective compounds can act on a high level of glutamate and on ROS, and can prevent neurodegenerative disorders.

Glutamate, an essential neurotransmitter of CNS at high concentration can cause ND. Several studies reported that neuronal cell death mediated by glutamate can cause various ND, including AD, brain trauma, cerebral ischemia, PD, epilepsy, and stroke [79–81]. High glutamate concentration results in excitotoxicity and high level production of reactive oxygen species (ROS), which further triggers neuronal cell death [82, 83]. It is thought that diseases associated with glutamatergic dysfunction produce disruption of calcium homeostasis, increased the production of nitric oxide and increases the oxidative stress resulting in programmed cell death and causing progressive neurodegeneration [79]. Regulating the glutamate levels can lower the excitotoxicity, ROS production and irregular influx of calcium may be an effectual therapeutic strategy for ND [84, 85]. Neuroprotective compounds have ability to inhibit glutamate-induced mitochondrial fission by regulating abnormal calcium influx and calcineurin-dependent dephosphorylation of Drp-1 through scavenging mitochondrial and cytosolic ROS [86]. Endophytic bioactive compounds such as ϒ-pyrone, fusarester D, karuquinone B, javanicin, solaniol, anhydrooxysproridinone, fischerin, and fusarubin, showed protective activity against glutamate induced cytotoxicity in *in vitro* models [87]. The proposed molecular mechanism of action of the neuroprotective compounds isolated from endophytes against glutamate induced neuronal cell death is presented in Figure 4.

Bang et al., identified total of nine bioactive compounds, namely, sartorypyrone E, sartorypyrone A, cyclotryprostatin B, fumitremorgin B, fumitremorgin A, aszonalenin, acetylaszonalenin, fischerin and pyripyropene A, by using IR, UV, ^1^H NMR, and ^1^C NMR techniques from the *Neosartorya fidcheri* JS0553 endophytic fungi isolated from *Glehnia littoralis* [87]. The protective effects of these bioactive compounds against HT22 cells were investigated on glutamate induced cytotoxicity. The result showed that among all the compounds, fischerin displayed the most significant neuroprotective effects in HT22 cell death induced with glutamate via inhibition of ROS, Ca^2+^, and phosphorylation of mitogen activated protein kinase (MAPKs) (via JNK, ERK1/2, and p38) [87]. In another study [88], five unique cyclic depsipeptides including colletotrichamide A, colletotrichamide B, colletotrichamide C, colletotrichamide D, and colletotrichamide E, with neuroprotective effects were isolated and identified from the endophytic fungi *Colletotrichum gloeosporioides* JS419 (inner tissue of *Suaeda japonica*). These compounds were tested for their protective effects against glutamate-induced HT22 cell death in which colletotrichamide B, colletotrichamide C, and colletotrichamide E showed protective effects, while colletotrichamide C displayed 100% viability (at 100 *µ*M) [88].

Bioactive compounds including alternin A, isosclerone, alternariol methyl ether, alternariol, stemphyperylenol, 1H-indole-3-carboxylic acid, indole-3-methylethanoate, ergosta-4,6,8(14),22-tetraen-3-one, (17R)-4-ydroxy-17-methylincisterol, (17R)-4-hydroxy-17-methylincisterol, (1R,5 R,6R,7 R,10S)-1,6-dihroxyeudesm-4(15)-ene, 3(*ζ*)-hydroxy-octadeca-4(E),6(Z)-dienoic acid, E-7,9-diene-11-methenyl palmitic acid, p-hydroxybenzonic acid, and benzoic acid, were isolated and identified through different spectroscopic analyses from *Alternaria alternate,* an endophytic fungi of *Psidium littorale*. These all 15 isolated compounds were tested against four different cancer cell lines such as 4T-1, A549, HepG-4, and MCF-7. Among all, only two compounds displayed significant cytotoxicity in terms of IC_50_ value [(17R)-4-hydroxy-17-methylincisterol: HepG-4 = 9.73 ± 1.2 *µ*M; stemphyperylenol: MCF-7 = 4.2 ± 0.6 *µ*M; HepG-4 = 7.9 ± 0.9 *µ*M]. Additionally, compound isosclerone, indole-3-methylethanoate, and (17R)-4-ydroxy-17-methylincisterol significantly improved the cell viability of glutamate-induced PC-12 cells from 67.8 ± 5.1% to 84.8 ± 6.5% at the concentration of 40 *µ*M and 80 *µ*M, respectively [89].

Several new bioactive compounds with different pharmacological potential have been isolated and identified from endophytic fungi of mangrove origin [90]. In this context, three unique polyketide-derived alkaloids (phomopsol A, B, and C) were isolated from the mangrove endophytic fungi *Phomopsis spp*., xy21 [91]. The compounds were determined using different spectroscopic analyses (XRD, NMR) and tested for their neuroprotective activity against PC12 cells. Among all three compounds, phomopsol A and phomopsol C showed neuroprotective effects in a dose dependent manner from 5.0 to 40.0 *µ*M, whereas cell viability was recorded as 76% (phomopsol A) and 96% (phomopsol C) at 40.0 *µ*M when compared with control (Corticosterone = 60% at 200.0 *µ*M) [91]. In another study, Wu and group [92] evaluated neuroprotective activities from compounds (Z)-7,4′-dimethoxy-6-hydroxy-aurone-4-O-*β*-glucopyranoside, and (1S,3 R,4S)-1-(4′-hydroxyl-phenyl)-3,4-dihydro-3,4,5-trimethyl-1H-2benzopyran-6,8-diol isolated from endophytic fungi *Penicillium citrinum* of mangrove tree *Bruguiera gymnorhiza*. The result suggested that (Z)-7,4′-dimethoxy-6-hydroxy-aurone-4-O-*β*-glucopyranoside displayed significant neuroprotective activity against MPP^+^ induced toxicity in PC12 cells and increases the cells viability. Additionally, it enhances the mitochondrial membrane potential, decrease the production of DNA fragmentation, and inhibited the caspase-3 and -9 in MPP^+^-treated PC12 cells [92].

Song et al. [19] isolated the endophytic fungi *Colletotrichum spp*., JS-0367 from *Morus alba* leaves and identified total of four antraquinones, namely, 1,3-dihydroxy-2,8-dimethoxy-6-methylanthraquinone, 1-hydroxy-2,3,8-trimethoxy-6-methylanthraquinone, 1,2-dihydroxy-3,8-dimethoxy-6-methylanthraquinone, and evariquinone by using spectroscopic analyses from it. All these compounds were tested against glutamate-induced HT22 cell death. Among these compounds, evariquinone displayed strong protective activity against glutamate-induced HT22 cell death via inhibition of intracellular ROS accumulation and Ca^2+^ influx. Additionally, evariquinone suppresses the phosphorylation of MAPKs (JNK, ERK1/2, and p38) induced by glutamate [19].

Inflammation is closely associated with the pathogenesis of ND such as AD, PD, multiple sclerosis, cerebral ischemia, and post-traumatic brain injuries. Harun and co-workers [93] investigated the role of five endophytic fungi extracts (HAB16R12, HAB16R13, HAB16R14, HAB16R18, and HAB8R24) against lipopolysaccharide-induced inflammatory events. In this study, all five extracts were investigated against nitric oxide (NO), CD40 phenotype, and pro- and anti-inflammatory cytokine production in LPS-BV2 microglia cells. The pretreatment of microglia cells with these extracts minimizes the NO production without affecting cell viability. These endophytic extracts significantly (*p* < 0.05) inhibited the expression of proinflammatory cytokines (IL-6 and TNF-alpha) in LPS produced by BV2 microglia. These neuroprotective effects of endophytic extracts are probably mediated via suppression of inflammation [93].

A number of endophytes (212) were isolated from the plants and evaluated for their BACE1 inhibitory activity by Harun and group [94]. Among all 212 endophytic extracts (1000 *µ*g/mL), only 29 endophytic extracts (HAB16R13, HAB16R18, HAB16R14, HAB8R24, HAB16R12, HAB6S14, HAB15R7, HAB16R15, KK9R1, HAB16R11, HAB6S11, HAB13S18, HAB4L5, HAB6R8, HAB4L3, KT36L1, HAB15R6, HAB16L32, KK11S3, KT39R1, HAB26S6, KT44S3, KT34L2, HAB8R19, HAB13L4, HAB13L2, HAB12S12, HAB13S13, and HAB13R29) displayed strong BACE1 inhibitory activity (more than 90%). Four extracts, namely, HAB16R13, HAB16R18, HAB16R14, and HAB8R24 showed IC_50 (BACE1)_ = 3.0 *µ*g/mL and the extract HAB16R13 IC_50 (BACE1)_ = 2.15 *µ*g/mL demonstrated the best BACE1 inhibitory activity among all. The most active endophytic extract (HAB16R13) was tested for cytotoxicity against PC-12 and WRL68 cells and the extract showed nonpotent cytotoxic effects in terms of IC_50 (CT)_ value (60 and 40 *µ*g/mL), respectively [94].

*Cistanche deserticola* (Y.C. Ma) is a popular medicinal plant of China used for the treatment of kidney deficiency and neurasthenia from a long time. An endophytic fungi *Penicillium chrysogenum* No. 005 were isolated from the roots of this species and was evaluated for bioactive compounds and their neuroprotective effects on oxidative stress-induced cell death in SH-SY5Y cells [95]. The total five compounds such as (1) chrysogenamide A, (2) circumdatin G, (3) 2-[(2′-hydroxypropionyl) amino] benzamide, (4) 2,3–dihydrosorbicillin, and (5) (9Z,12Z)-2,3-dihydroxypropyloctadeca-9,12-dienoate were isolated and identified by using NMR analysis. The compound 1 did not show any significant ability (IC_50_: >100 *µ*M) to scavenge DPPH-free radicals up to 100 *µ*M concentration when compared with the control (ascorbic acid: IC_50_: >29.0 *µ*M), whereas compound 1 showed neuroprotective activity against oxidative stress induced by hydrogen peroxide by improving cells viability up to 59.6% (1 × 10^−4^ *µ*M) [95]. A detailed description of endophyte compounds against neurological diseases is presented in Table 1.

## 5. Conclusion and Future Prospects

The global diversity of endophytic fungi is far from being accessed, and these endophytic fungi are considered as a metabolic factory capable of unique bioactive compound production. This type of chemical diversity is important for the screening of novel bioactive compounds targeting different types of diseases, which allows them to act as a prototype compound for the development of new specific drugs. The present manuscript is focused on describing “endophytic fungi as a source of bioactive compounds and their *in-vitro* neuroprotective activities.” The literature survey clearly demonstrated that endophytic fungi and their bioactive compounds played an important role in neuroprotective studies via different pathways, and showed significant results. Furthermore, the isolated active compounds need to be elucidated and authenticated by *in-vivo* studies as well as clinical studies. Since most of the reported studies are limited to the *in-vitro* screening, future clinical trials should be conducted to assess the safety issues of these bioactive compounds in the human body in terms of different biological activities.

## Figures and Tables

**Table 1:** Neuroprotective effects of few bioactive compounds isolated from endophytes.

S.N.	Endophytic fungi	Isolated compounds from endophytes	Neuroprotective effects	References
1.	*Nigrospora oryzae*	Quercetin and (GL15) isolates	↓AChE	[71]
2.	*Alternaria alternate*	Alternariol, alternariol 5-O-methyl ether, altenusin B, altenuene, altenusin, alterlactone and dehydroaltenusin	↑Nirf-2	[72]
3.	*Fusarium spp.*	OQ-fus-2-F	↓AChE	[73]
4.	*Chaetomium globosum* and *Phomopsis spp*.	Chaetoglobosin F, chaetoglobosin fex, chaetoglobosin E, cytoglobosin A, penochalasin C, isochaetoglobosin D, cytochalasin H, and 18-methoxycytochalasin J	↓H_2_O_2_/MMP^+^, ↓Lactate dehydrogenase	[74]
5.	*Fusarium lateritium*	6-deoxyoysporidinone (SSF2-1), 4,6′-anhydrooxysproridinone (SSF2-2), and sambutoxin (SSF2-3)	↓ROS, ↑O^2−^, ↓Ca^2+^ influx, ↑Nrf2, ↓cytochrome c	[75]
6.	*Fusarium solani*	ϒ-pyrone, fusarester D, karuquinone B, javanicin, solaniol, and fusarubin	↑Cell viability	[76]
7.	*Alternaria brassicae*	Huperzine A, AGF040 to AGF050	↓AChE	[78]
8.	*Neosartorya fidcheri*	Sartorypyrone E, sartorypyrone A, cyclotryprostatin B, fumitremorgin B, fumitremorgin A, aszonalenin, acetylaszonalenin, fischerin, and pyripyropene A	↓ROS, ↓Ca^2+^, ↓MAPKs	[87]
9.	*Colletotrichum gloeosporioides*	Colletotrichamide A, colletotrichamide B, colletotrichamide C, colletotrichamide D, and colletotrichamide E	Protective effects against glutamate induced HT22 cell death	[88]
10.	*Colletotrichum spp.*	1,3-dihydroxy-2,8-dimethoxy-6-methylanthraquinone, 1-hydroxy-2,3,8-trimethoxy-6-methylanthraquinone, 1,2-dihydroxy-3,8-dimethoxy-6-methylanthraquinone, and evariquinone	↓ROS, ↓Ca^2+^, ↓MAPKs	[19]

## Data Availability

All data are included within the text.
